# Comparative evaluation of VISTA using platelet-rich fibrin and placental membrane for multiple gingival recession

**DOI:** 10.6026/973206300214837

**Published:** 2025-12-15

**Authors:** Nandini R, Ramesh Amirisetty, Lakshmi Anusha Dornipati, B. Ravinder Reddy, Neha Singh, Tanuja B

**Affiliations:** 1 Department of Periodontology, G. Pulla Reddy Dental College and Hospital, Kurnool, Andhra Pradesh, India; 2 Department of Dental Surgery, RDH, Florida, US; 3 Department of Periodontology, Tirumala Institute of Dental Sciences, Nizamabad, Telangana, India

**Keywords:** Gingival recession, Vestibular incision subperiosteal tunnel access (VISTA) technique, platelet-rich fibrin, placental membrane, root coverage, periodontal plastic surgery

## Abstract

Gingival recession is a prevalent clinical problem and limited evidence exists comparing biological membranes used with the VISTA technique for its correction. Therefore, it is of interest to evaluate the effectiveness of VISTA combined with Platelet-Rich Fibrin (PRF) versus placental membrane in 20 sites with Miller's Class I/II recession. Both groups demonstrated significant improvement in all clinical parameters-including RD, RW, CAL, WKG and GT-over a 6-month period (p<0.001). Intergroup comparisons revealed no statistically significant differences between PRF and placental membrane across all evaluated variables. Both biomaterials were equally effective when combined with the VISTA technique, supporting their interchangeable use in managing multiple gingival recessions.

## Background:

Gingival recession, defined as the displacement of marginal tissue apical to the cementoenamel junction, is a common clinical finding affecting more than 50% of the population, with buccal areas being most frequently affected [1]. This condition presents both functional and aesthetic challenges, often leading to root hypersensitivity, root caries and compromised appearance [2]. The etiology of gingival recession is multifactorial, including plaque-induced inflammation, improper oral hygiene practices, tooth malposition, high frenum attachment and anatomical factors [3]. The management of gingival recession has evolved significantly over the past decades. Initially termed "mucogingival surgery" by Friedman in 1957, the field transformed into "periodontal plastic surgery" as described by Miller in 1993, focusing on correcting anatomic, developmental, or disease-induced defects of the gingiva and alveolar mucosa [4]. The ultimate goal of root coverage procedures is complete coverage of the recession defect with minimal probing depth and optimal aesthetic appearance [5]. Various classification systems have been proposed to facilitate diagnosis and treatment planning. Miller's classification (1985) remains widely used, categorizing recessions into four classes based on the extent of tissue loss and interdental bone involvement [6]. More recently, Cairo *et al.* (2011) introduced a classification based on interproximal clinical attachment loss, providing a more comprehensive approach to prognosis determination [7]. Multiple surgical techniques have been developed for root coverage; including pedicle flaps, free gingival grafts and regenerative procedures [8]. The Vestibular Incision Subperiosteal Tunnel Access (VISTA) technique, introduced by Zadeh in 2011, represents a minimally invasive approach for treating multiple gingival recessions [9]. This technique offers advantages including easy access, trauma reduction and maintenance of vascular supply and coronal repositioning of the gingival margin [10].

Biomaterials play a crucial role in enhancing outcomes of periodontal plastic surgery. Platelet-Rich Fibrin (PRF), is a second-generation platelet concentrate that promotes healing through the sustained release of growth factors and cytokines [11]. PRF contains platelets, leukocytes and various growth factors including PDGF, TGF-β, VEGF and IGF, which stimulate angiogenesis, mitogenesis and tissue regeneration [12]. Placental membranes, particularly amniotic membrane, have emerged as versatile biomaterials in periodontal regeneration. First used in skin transplantation amniotic membrane possesses inherent biological properties including anti-inflammatory, antimicrobial and anti-scarring effects [13]. It contains collagen types I, III, IV, V and VI, along with growth factors that promote epithelial cell migration and adhesion [14]. Additionally, amniotic membrane exhibits low immunogenicity, making it suitable for allogeneic transplantation [15]. While both PRF and placental membrane have shown promising results in periodontal regeneration, limited comparative studies exist evaluating their efficacy when used with the VISTA technique. A study by Shetty *et al.* (2014) compared PRF with amniotic membrane in bilateral multiple recession coverage, finding both materials effective [16]. However, no studies have directly compared these biomaterials when used specifically with the VISTA technique for treating multiple gingival recessions. Therefore, it is of interest to evaluate and compare the effectiveness of the VISTA technique using either PRF or placental membrane in the treatment of Class I or II multiple gingival recession.

## Materials and Methods:

## Study design and sample size:

A randomized clinical trial was conducted at the Department of Periodontics and Implantology, G. Pulla Reddy Dental College and Hospital, Kurnool andhra Pradesh. The study included 20 surgical sites from patients with multiple gingival recessions. Based on previous similar studies, a sample size of 10 sites per group was determined to provide 80% power to detect significant differences at α=0.05.

## Selection criteria:

## Inclusion criteria:

[1] Systemically healthy patients aged between 20-50 years

[2] Presence of Miller's Class I or II gingival recession defects

[3] Anterior teeth and premolars affected

[4] Good oral hygiene maintenance (Plaque Index <1.5)

[5] Non-smokers

## Exclusion criteria:

[1] Systemic diseases including diabetes

[2] Pregnant or lactating mothers

[3] History of periodontal surgery in the same area within six months

[4] Patients with poor compliance

[5] Active periodontal infection

## Group allocation:

After initial periodontal therapy, 20 surgical sites were randomly divided into two groups:

[1] Group A (n=10): Treated with VISTA technique using Platelet-Rich Fibrin membrane

[2] Group B (n=10): Treated with VISTA technique using Placental (Amniotic) membrane

## Clinical parameters:

The following clinical parameters were recorded at baseline, 1 month, 3 months and 6 months post-operatively using a prefabricated acrylic stent for standardization:

[1] Plaque Index (PI): Assessed using Silness and Loe (1964) criteria

[2] Gingival Index (GI): Evaluated using Loe and Silness (1963) criteria

[3] Recession Depth (RD): Measured at the middle of the buccal surface from the cementoenamel junction to the crest of the free gingival margin

[4] Recession Width (RW): Measured across the buccal surface at the cementoenamel junction level

[5] Clinical Attachment Level (CAL): Measured mid-buccally from the cementoenamel junction to the base of the periodontal pocket

[6] Width of Keratinized Gingiva (WKG): Measured from the most apical point of gingival margin to the mucogingival junction

[7] Gingival Thickness (GT): Measured 2-3 mm below the gingival margin at the attached gingiva using an endodontic spreader with a stopper, with the distance measured using digital Vernier calipers

## Surgical procedure:

## Pre-surgical preparation:

All patients received comprehensive oral hygiene instructions and underwent non-surgical periodontal therapy. Acrylic stents were fabricated for each patient with grooves for standardized measurements.

## PRF preparation:

For Group A, 20 ml of intravenous blood was drawn from each patient into two sterile test tubes and immediately centrifuged at 3000 rpm for 10 minutes. The PRF clot was separated from the red blood cell base and placed in a PRF box to obtain a membrane.

## Placental membrane preparation:

For Group B, commercially available freeze-dried amniotic membrane was rehydrated according to manufacturer instructions before use.

## VISTA surgical technique:

Under local anesthesia (2% lignocaine with 1:80,000 adrenaline), the following steps were performed:

[1] Root conditioning with tetracycline solution followed by saline rinse

[2] Vestibular access incision using a 15C blade

[3] Creation of subperiosteal tunnel extending at least 1-2 teeth beyond the recession area using periosteal elevators

[4] Tunnel elevation beyond the mucogingival junction and interproximally under papillae

[5] Placement of either PRF membrane (Group A) or placental membrane (Group B) in the tunnel, extending 3-5 mm beyond the bony dehiscence

[6] Coronally advanced positioning of the mucogingival complex

[7] Stabilization using 4.0 polypropylene suture with coronally anchored technique secured with composite resin

[8] Closure of access incision with interrupted sutures

[9] Application of non-eugenol periodontal dressing (Coe-pack)

## Postoperative care:

All patients received antibiotics (amoxicillin 500mg TID for 5 days) and analgesics (ibuprofen 400mg TID for 3 days). Patients were instructed to avoid brushing the surgical area for two weeks. Sutures were removed after 14 days and postoperative evaluations were performed at 1, 3 and 6 months.

## Statistical analysis:

Data were analyzed using SPSS version 22.0 (IBM Corp., Armonk, NY, USA). Descriptive statistics were expressed as mean ± standard deviation (SD). Intragroup comparisons were made using Friedman test and Wilcoxon sign rank test for non-parametric data and ANOVA and paired t-test for parametric data. Intergroup comparisons were performed using Independent t-test and Mann-Whitney U test. A p-value <0.05 was considered statistically significant.

## Results:

The study included 20 surgical sites from 20 patients (12 females, 8 males) with a mean age of 32.4±7.8 years. No significant differences were observed between groups regarding age, gender distribution, or baseline clinical parameters (p>0.05). The most commonly affected teeth were maxillary anterior teeth (45%) followed by mandibular anterior teeth (35%) and premolars (20%). Both groups maintained good oral hygiene throughout the study period. In Group A, PI decreased from 0.98±0.23 at baseline to 0.81±0.18 at 6 months, while GI decreased from 1.08±0.24 to 0.90±0.16. In Group B, PI decreased from 1.07±0.32 to 0.91±0.23 and GI decreased from 1.16±0.31 to 0.92±0.22. These changes were not statistically significant within or between groups (p>0.05). Both groups showed significant improvements in all clinical parameters from baseline to 6 months (p<0.001). Table 1 (see PDF) presents the intragroup comparison of clinical parameters at different time intervals. Table 2 (see PDF) shows the mean differences in clinical parameters from baseline to 6 months for both groups. The percentage of root coverage achieved was 62.5% in Group A and 58.7% in Group B. Table 3 (see PDF) presents the intergroup comparison of clinical parameters based on mean differences from baseline to 6 months. No statistically significant differences were observed between the two groups for any parameter (p>0.05).

## Discussion:

This study evaluated and compared the effectiveness of VISTA technique using either PRF or placental membrane in treating multiple gingival recessions. Both groups showed significant improvements in all clinical parameters from baseline to 6 months, with no statistically significant differences between groups. The significant reduction in recession depth and width observed in both groups can be attributed to several factors. The VISTA technique itself offers advantages including minimal trauma, maintenance of vascular supply and coronal positioning of the gingival margin [9]. Zadeh (2011) reported complete root coverage and gain in keratinized gingiva using VISTA with collagen membrane and growth factors, supporting our findings [9]. Additionally, the selection of recession defects less than 4mm in our study likely contributed to favorable outcomes, as larger defects are associated with lower predictability of complete root coverage [17]. The biomaterials used in this study also played a crucial role in the observed improvements. PRF, a second-generation platelet concentrate, promotes healing through the sustained release of growth factors including PDGF, TGF-β, VEGF and IGF [11]. These factors stimulate angiogenesis, mitogenesis and tissue regeneration, explaining the significant improvements in clinical parameters observed in Group A [12]. Our findings align with those of Subbareddy *et al.* (2020), who reported significant improvements in clinical parameters when using VISTA with PRF for multiple gingival recessions [18]. Similarly, placental membrane contains various growth factors, cytokines and extracellular matrix components that enhance wound healing and tissue regeneration [13]. The amniotic membrane's anti-inflammatory, antimicrobial and anti-scarring properties, along with its low immunogenicity, make it an excellent biomaterial for periodontal regeneration [14]. Our results in Group B are consistent with those of Rehen *et al.* (2018), who found significant improvements in clinical parameters when using amniotic membrane with coronally advanced flap for recession coverage [19]. The significant increase in width of keratinized gingiva observed in both groups is clinically important, as an adequate zone of keratinized tissue is essential for maintaining periodontal health [16, 17, 18, 19-20]. The increase in gingival thickness is also noteworthy, as thicker gingival tissue is associated with better aesthetic outcomes and resistance to recession [21]. Both PRF and placental membrane appear to promote these favorable tissue changes, though through different mechanisms. The lack of significant differences between groups suggests that both biomaterials are equally effective when used with the VISTA technique. This finding has clinical implications, as the choice between PRF and placental membrane may depend on factors such as cost, availability and patient preference rather than efficacy. PRF offers the advantage of being autologous and cost-effective, while placental membrane eliminates the need for blood collection and processing [22]. The relatively small sample size and short follow-up period (6 months) may limit the generalizability of our findings. Additionally, we did not perform histological analysis to confirm the nature of tissue regeneration. Future studies with larger sample sizes, longer follow-up periods and histological evaluation are warranted to further validate our findings.

## Conclusion:

Both PRF and placental membrane when used with the VISTA technique are equally effective in treating multiple gingival recessions. Significant improvements were observed in all clinical parameters including recession depth, recession width, clinical attachment level, width of keratinized gingiva and gingival thickness in both groups. The choice between these biomaterials may depend on factors such as cost, availability and patient preference rather than efficacy. Further long-term studies with larger sample sizes are recommended to confirm these findings.

## References

[1] Santamaria MP (2025). Clin Adv Periodontics..

[2] Hegde S (2021). J Indian Soc Periodontol..

[3] Jain KS (2021). J Indian Soc Periodontol..

[4] Al-Barakani MS (2024). Head Face Med..

[5] Boddu T (2023). Int J Periodontics Restorative Dent..

[6] Miller PD (1985). Int J Periodontics Restorative Dent..

[7] Cairo F (2011). J Clin Periodontol..

[8] Zucchelli G (2015). Periodontol 2000..

[9] Zadeh HH (2011). Int J Periodontics Restorative Dent..

[10] Kim S (2025). J Periodontal Implant Sci..

[11] Dohan DM (2006). Oral Surg Oral Med Oral Pathol Oral Radiol Endod..

[12] Miron RJ (2017). Clin Oral Investig..

[13] Niknejad H (2008). Eur Cell Mater..

[14] Zucchelli G, De Sanctis M (2000). J Periodontol..

[15] Kuis D (2013). J Periodontol..

[16] Shetty SS (2014). J Indian Soc Periodontol..

[17] Holbrook T (1983). Int J Periodontics Restorative Dent..

[18] Subbareddy BV (2020). Contemp Clin Dent..

[19] Rehen M (2018). Contemp Clin Dent..

[20] Wennström  JL, Zucchelli G (1996). J Clin Periodontol..

[21] Castellanos A (2006). J Periodontol..

[22] Cheng YF (2007). J Periodontal Res..

